# COvid MEdicaTion (COMET) study: protocol for a cohort study

**DOI:** 10.1136/ejhpharm-2020-002329

**Published:** 2020-06-25

**Authors:** Roos S G Sablerolles, Freija E F Hogenhuis, Melvin Lafeber, Bob P A van de Loo, Sander D Borgsteede, Eric Boersma, Jorie Versmissen, Hugo M van der Kuy, COMET Research team, Jacomien Aleman

**Affiliations:** 1 Department of Internal Medicine, Erasmus MC University Medical Center, Rotterdam, The Netherlands; 2 Department of Hospital Pharmacy, Erasmus MC University Medical Center, Rotterdam, The Netherlands; 3 Digitalis Rx BV, Amsterdam, The Netherlands; 4 Department of Clinical Decision Support, Health Base Foundation, Houten, Utrecht, The Netherlands; 5 Department of Cardiology, Erasmus MC University Medical Center, Rotterdam, The Netherlands

**Keywords:** hypertension, virology, clinical pharmacy, adverse effects, vascular medicine

## Abstract

Various theories about drugs such as ACE inhibitors or angiotensin II receptor blockers (ARBs) in relation to severe acute respiratory syndrome coronavirus 2 (SARS-CoV-2) and clinical outcomes of COVID-19 are circulating in both mainstream media and medical literature. These are based on the fact that ACE2 facilitates SARS-CoV-2 cell invasion via binding of a viral spike protein to ACE2. However, the effect of ACE inhibitors, ARBs and other drugs on ACE2 is unclear and all theories are based on conflicting evidence mainly from animal studies. Therefore, clinical evidence is urgently needed. The aim of this study is to investigate the relationship between use of these drugs on clinical outcome of patients with COVID-19. Patients will be included from several hospitals in Europe. Data will be collected in a user-friendly database (Digitalis) on an external server. Analyses will be adjusted for sex, age and presence of cardiovascular disease, hypertension and diabetes. These results will enable more rational choices for randomised controlled trials for preventive and therapeutic strategies in COVID-19.

## Introduction

Understanding the pathophysiology of the COVID-19 pandemic is key to develop strategies for both prevention and treatment to improve clinical outcomes. It is known from the previous outbreak of severe acute respiratory syndrome coronavirus (SARS-CoV) that cell invasion is accomplished by binding of a viral spike protein to ACE2.^1^ Recent reports show that this is the same for SARS-CoV-2.^2 3^ This binding leads to ACE2 downregulation, which may contribute to lung injury, since less ACE2 is available for potential protective effects, as shown in acute respiratory distress syndrome (ARDS).^4^

ACEs are regulatory proteins in both blood pressure and inflammation. ACE1, the key enzyme in the renin–angiotensin aldosterone system (RAAS), converts angiotensin I to angiotensin II which by binding to the angiotensin II receptor type 1 leads to vasoconstriction, resulting in a rise of blood pressure, fibrotic and proliferative/inflammatory effects. ACE1 is targeted for reducing blood pressure by ACE inhibitors; angiotensin II receptor blockers (ARBs) lead to similar effects by blocking the effects of angiotensin II.

ACE2 degrades angiotensin I to angiotensin-(1-9) and angiotensin II to angiotensin-(1-7)(4). This pathway involving the Mas receptor is thought to be counteracting the effects of angiotensin II and is associated with protective effects for heart, lungs and kidneys.^5^ However, studies targeting this pathway have not led to beneficial effects in cardiovascular and renal diseases including hypertension.^6 7^ In addition to its role in RAAS modulation, ACE2 is also involved in degrading several other substrates, such as apelin, bradykinin and opioids. Recently, involvement in degrading bradykinin has also been suggested as playing a causal role in the development of severe ARDS, since downregulation of ACE2 as a consequence of virus binding may enhance the proinflammatory bradykinin receptor leading to local vascular leakage.^8^

The fact that patients with previous cardiovascular disease (CVD), hypertension and diabetes have an increased mortality risk due to SARS-CoV-2-induced infection could be explained by the common factor of use of ACE inhibitors or ARBs.^9 10^ This was suggested by several authors and also reached the mainstream media including *CNN*.^11 12^ However, well-designed studies on the effects of ACE inhibitors, ARBs and non-steroidal anti-inflammatory drugs on ACE2 expression are scarce, show conflicting results and have not been conducted in humans.^6 13–15^ This has led to statements from several societies, including the European Society of Cardiology and the European Society of Hypertension, emphasising the lack of evidence and the importance of not withdrawing ACE inhibitors and ARBs considering their major role in blood pressure lowering, nephroprotection and cardiac protection.^5^ In reply to these first rumours, other authors, including Versmissen *et al*, argued that RAAS inhibition might even be beneficial once infected.^16–18^ Other authors suggested that ARBs might be beneficial while ACE inhibitors might not.^19^

Based on these theoretical grounds, intervention studies, either starting or discontinuing ACE inhibitors or ARBs, are currently recruiting patients with COVID-19 (ClinicalTrials.gov). However, observational data in humans are urgently needed to address the real effect of medication and the clinical course of COVID-19 before starting potentially harmful interventions based on only theoretical and conflicting evidence from animal studies. Therefore, the COMET study aims to evaluate the relationship between use of certain drugs, starting with ACE inhibitors and ARBs, on clinical outcomes of patients with COVID-19. The main objective is to describe the correlation between use of ACE inhibitors or ARBs and clinical outcome, defined by admission to the hospital, duration of stay, intensive care unit (ICU) admission and survival, in patients with confirmed COVID-19. Secondary aims are to address the effect of other drugs, such as immunomodulatory agents, including non-steroidal anti-inflammatory drugs and corticosteroids.

## Methods and analysis

An observational cohort study has been designed. Patients will be included from various hospitals in Europe by a pharmacist, clinical pharmacologist or treating physician. The participating investigators are asked to consecutively include all patients registered at the emergency department (ED) on a certain day or several days until a minimum of 50 patients have been included. If ED registration is incomplete, all patients with COVID-19 who are hospitalised during a specific period until a minimum of 50 patients has been reached (both ICU and normal ward) will be entered. The major criterion for a patient to be included is COVID-19 positive by a positive SARS-CoV-2 PCR or high clinical likelihood based on bilateral pulmonary infiltrates not explained by another cause or after consensus of the local COVID-19 expert team based on clinical, biochemical and radiological criteria.

### Data collection

To allow urgent analysis, data entry will be undertaken in two phases. For quick data entry, the number of collected parameters will be minimised and with a special focus on medication. The following subject parameters will be collected for all patients: year of birth, sex, International Classification of Primary Care/International Classification of Diseases, 10th Revision medical history including CVD, diabetes, hypertension, body mass index (normal 20–25 kg/m^2^, high 25–30 kg/m^2^ or obese >30 kg/m^2^), prescribed medication by Anatomical Therapeutic Chemical code and dose (at moment of presentation), smoking history, clinical frailty score,^20^ logistic course (presentation at ED/transmission from/to another hospital), clinical course (no admission/admission to hospital <48 hours/>48 hours, no ICU, admission ICU) and survival. If easily accessible, laboratory data at presentation will also be entered. In the second phase, additional parameters may be included such as more specific clinical outcomes such as pulmonary embolism or more extensive laboratory parameters.

### Definitions and clinical outcomes

Since the medical history may be incomplete at the ED as recorded by the collaborating researchers, medication data will be used to confirm CVD, diabetes, heart failure and hypertension or to correct when medical history appears to be incomplete. CVD, including coronary artery disease, cerebrovascular disease and peripheral arterial occlusive disease, will be considered present when entered as items in the medical history and when a patient is using antiplatelet therapy to prevent bias of misdiagnosed CVD, especially in the case of angina.

Diabetes will be considered present when antidiabetic medication is used, even in the absence of a note in the medical file, to prevent bias of undiagnosed diabetes. Hypertension will be considered present when entered as an item in the medical history and antihypertensive medication is used or when a patient is using one or more antihypertensive drugs without other indication. Heart failure will be considered present when entered as an item in the medical history or when a patient is using an ACE inhibitor or ARB, a beta blocker and a loop diuretic in the absence of severe renal insufficiency.

### Outcome

Clinical outcome will be assessed during 2 weeks after the date of COVID-19 diagnosis, and scored as no hospital admission, hospital admission <48 hours, hospital admission >48 hours without ICU, hospital admission >48 hours with ICU. In addition, survival data will be collected.

### Data analysis

The relation between ACE inhibitors/ARBs and clinical outcomes will first be assessed by univariate and multivariate logistic regression analyses. Adjustments will be made for the following potential confounding factors: sex, age and presence of CVD, diabetes and hypertension. A dose–response analysis will be performed to support a causal relationship.

Since only patients visiting the ED will be included, they might not be representative for the larger population not visiting the hospital. Therefore, the aim is to compare the use of ACE inhibitors/ARBs in the Dutch patients with the general Dutch population of similar age (age group of 10 years; Stichting Farmaceutische Kengetallen: foundation pharmaceutical key numbers). In addition, use of other blood pressure-lowering agents and other drug classes such as lipid-lowering drugs will be compared between patients with most severe outcomes and no hospital admission or a short stay (<48 hours).

## Ethics and dissemination

The medical ethical committee of Erasmus MC and Brabant concluded this research does not fall under the scope of the Medical Research Involving Human Subjects Act (non-WMO) because of its retrospective nature and full anonymisation. Investigators from each hospital will fill in anonymised data (sex and birth year) and all will sign a data transfer agreement. Each patient will be assigned a study number known only by the investigator who will keep a coding list in the own hospital. Each hospital can only see their own patients in the system. Data will be collected in a database (digitalis.nl) on an external server and then stored using Open Clinica. All data will be treated according to the privacy regulations applicable for Europe and the Erasmus MC privacy regulations. Collected data will be secured against unauthorised access and will be stored and secured by the department of Hospital Pharmacy. No data that can identify a patient will be processed on this database to protect and respect the privacy of all patients. The main research team including the principal investigator can see all anonymised data. The main article will be submitted to a general medicine journal; follow-up articles focusing more deeply on certain drug classes might be submitted to pharmacological journals or journals in the specific area of disease, for instance cardiovascular.

## Discussion

Currently, clinical trials targeting ACE2, RAAS and the bradykinin pathway are initiated while the evidence that this might be beneficial is still weak. The results of this study may contribute to more rational choices for randomised controlled trials. In addition, when any negative effect of use of ACE inhibitors or ARBs is identified, changing medication might be an easy intervention to reduce morbidity and mortality from COVID-19. For instance ACE inhibitors and ARBs are used by over 10% of the Dutch population (gipdatabank.nl); almost half of these users are aged above 70 years.

The most serious limitation of this study is the selection bias, especially when ED registration of unhospitalised patients is incomplete. However, for comparing ACE inhibitors and ARBs the indications are similar preventing a bias by indication; in addition, other antihypertensive drugs and lipid-lowering drugs will be analysed. Due to the urgency of need of this data, time to enter clinical data will be limited, potentially leading to incomplete data, especially the medical history. However, by using detailed medication data this can largely correct for incomplete medical history data.
